# Pediatric scalp, face and neck burns in a referral pediatric burn centre in Türkiye

**DOI:** 10.3389/fped.2026.1896555

**Published:** 2026-08-24

**Authors:** Zeliha Akış Yıldız, Sabri Demir

**Affiliations:** 1 Children’s Hospital Department of Pediatric Surgery Pediatric Burn Center, Ankara Bilkent City Hospital, Ankara, Türkiye; 2 Children’s Hospital Department of Pediatric Surgery Pediatric Burn Center, University of Health Sciences Ankara Bilkent City Hospital, Ankara, Türkiye

**Keywords:** face burns, morbidity, neck burns, pediatric burns, scalp burns

## Abstract

Burns affecting the head, face, and neck region present challenging anatomical, functional, and aesthetic consequences. However, data comparing these injuries with other burn areas are scarce. This study aimed to evaluate and compare the epidemiological and clinical characteristics of pediatric patients with isolated scalp, face, and neck burns, those with scalp, face, and neck burns involving additional body regions, and those without scalp, face, and neck involvement. Following Ethics Committee approval, pediatric patients (0–18 years) admitted to tertiary burn centers between 1August 2019 and July 31, 2025, were retrospectively analyzed. Patients were divided into three groups: isolated head, face, and neck burns (group 1) (*n* = 88), head, face, and neck burns with burns in other areas (group 2) (*n* = 569), and burns without head, face, and neck involvement (group 3) (*n* = 822). Demographic characteristics, burn etiology, total body surface area, inhalation injury, need for intubation, length of hospital stay, and mortality were compared. Statistical significance was set at *P* < 0.05. A total of 1,479 patients were included in this study. Patients in Group 2 had larger burn areas. Respiratory tract injury (8.8%) and the need for intubation (10.0%) were more frequent in group 2 than in the other groups (*P* < 0.001). The overall mortality rate was 2.0%. The highest mortality was observed in patients in Group 2 (3.2%). No mortality was observed in group 1. The results of our study showed that head, face, and neck burns alone do not increase morbidity and mortality in children; however, when combined with burns in other areas, there is a likely increase in morbidity and mortality due to increased total body surface area and inhalation injury.

## 1 Introduction

Burn injuries in children are a significant global public health challenge. Although such injuries are largely preventable, they remain a leading cause of trauma-related morbidity and mortality among children, particularly in low- and middle-income countries (1–3).

In pediatric patients, burns involving the scalp, face, and neck (SFN) represent a distinct clinical entity due to their anatomical complexity and the substantial functional and psychosocial impact associated with burns in these anatomical regions. The face plays a critical role in airway protection, physical appearance, communication and social interaction, so burns involving this region present unique therapeutic challenges. Children with SFN burns may experience serious problems such as aesthetic concerns, loss of self-esteem, voice and speech limitations, hearing dysfunction due to ear loss and difficulty wearing glasses. SFN burns are also often associated with inhalation injury. Specifically, in the acute phase, complications such as airway obstruction, acute respiratory distress and pneumonia can necessitate prolonged mechanical ventilation or even result in death. In the long term, serious respiratory complications (e.g., subglottic stenosis, speech impairment and impaired respiratory function) may occur (4, 5).

Although substantial epidemiological studies of pediatric burn victims have been performed, there are limited region-specific data, particularly concerning SFN burns, in the literature (1, 6–10). Studies reporting detailed demographic data, burn mechanisms and clinical outcomes related to SFN burns can aid the development of strategies for preventing burns and improve acute treatment and long-term outcomes.

Our Pediatric Burn Center is a referral hospital that accepts patients from surrounding Turkish provinces and abroad. To provide a comprehensive overview of pediatric scalp, face, and neck (SFN) burns in Türkiye, this study aimed to compare the epidemiological and clinical characteristics and outcomes of patients with isolated SFN burns, SFN burns involving additional body regions, and burns without SFN involvement, and to examine age-related differences in burn etiology and mechanisms of injury among patients with SFN involvement.

## 2 Materials and methods

### 2.1 Study design and population

This study was conducted on pediatric burn patients. Institutional review board approval (TABED 1-26-2172; February 11, 2026) was obtained before the commencement of the study. All pediatric patients aged 0–18 years who were hospitalized at the Pediatric Burn Center between August 1, 2019, and July 31, 2025, were included in the study. Patient files were retrospectively reviewed, and the patients' demographic and clinical data were recorded.

**Figure 1:**
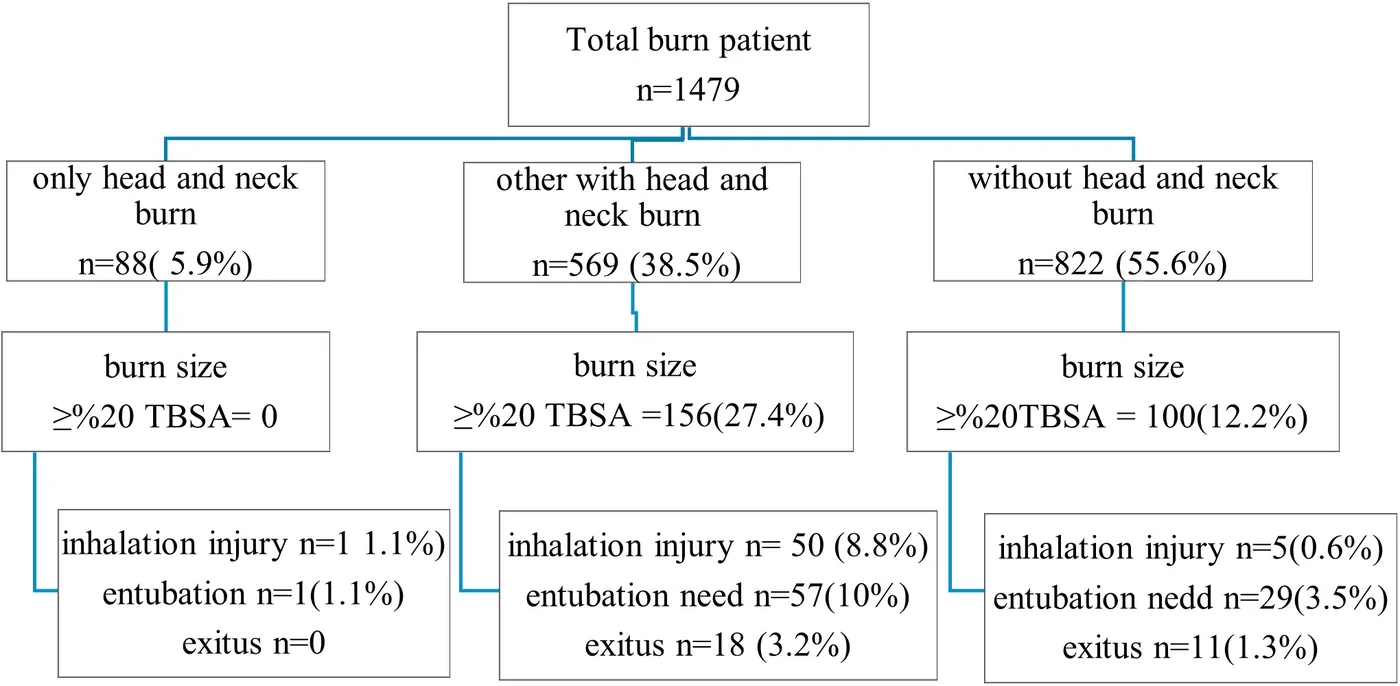
Flow diagram of the study population according to scalp, face and neck burn involvement. The study population included 1,479 pediatric burn patients. Patients were categorized into three groups: isolated scalp, face and neck burns, scalp, face and neck burns with additional body region involvement, and burns without scalp, face and neck involvement. Burn size >20% total body surface area, inhalation injury, intubation requirement, and mortality are summarized for each group.

The patients were divided into three groups: burns only on SFN (Group 1), SFN burns combined with burns in other areas (Group 2) and burns not affecting SFN (Group 3) (Figure 1). The groups were compared in terms of age, sex, nationality, percentage of burns (total body surface area: TBSA), lengh of hospital stay (LOS), presence of inhalation injury, need for mechanical ventilation and mortality. In addition, in patients with SFN burns classified as Group 1 and 2, age-related differences in sex distribution, burn etiology, and burn mechanism were analyzed.

Five age groups were established: infant (<1 year), toddler (2–3 years), preschool (4–5 years), school age (6–10 years) and adolescent (11–18 years). TBSA was calculated according to the Lund–Browder card (11, 12). Patients were divided into three groups according to TBSA burned: <20%, 20%–49% and ≥50%.

Burn depth was classified as second-degree or third-degree; however, the TBSA calculation was based on the total burned surface area regardless of burn depth.

In patients clinically suspected of having inhalation injury, bronchoscopy was performed to confirm the diagnosis. Intubation was performed in accordance with the standard airway protocols.

### 2.2 Statistical analysis

Statistical analyses were performed using the Statistical Package for Social Sciences (SPSS) software version 21 (SPSS Inc., Chicago, IL, USA). Numerical variables, age and LOS are expressed as mean and standard deviation (SD). The normality of the distribution of these variables was investigated using the Kolmogorov–Smirnov test. Continuous variables with a normal distribution were compared using analysis of variance (ANOVA). Categorical variables were expressed as numbers and percentages. The significance of differences between groups was investigated using the chi-square test. *P* < 0.05 was considered to indicate statistically significant differences. When differences were found to be significant upon comparing more than two categorical groups, pairwise comparisons were performed with Bonferroni correction to determine which groups differed from each other. To identify factors independently associated with mortality, a multivariable binary logistic regression analysis was performed.

## 3 Results

Between August 1, 2019, and July 31, 2025, 1,479 patients were admitted and treated at our pediatric burn center. The mean age of the patients was 5.33 ± 5.17 years, and 909 (61.5%) were males. There were 88 patients in group 1 (5.9%), 569 in group 2 (38.5%), and 822 in group 3 (55.6%). Although there were more males in all groups, no significant differences were found between the groups in terms of age and sex (Table 1).

**Table 1:** Demographic and clinical characteristics of pediatric patients with burns according to burn region involvement.

	Group1	Group2	Group 3	All patients	*P* value
	*n* = 88	*n* = 569	*n* = 822	*n* = 1,479	
Age (years), mean (±SD)	5.58 (5.29)	5.52 (5.72)	5.18 (4.75)	5.33 (5.17)	0.42
Sex *n* (%)					0.82
Male	53 (60.2)	370 (65.0)	486 (59.1)	909 (61.5)	
Female	35 (39.8)	199 (35.0)	336 (40.9)	570 (38.5)	
TBSA (%)					0.001
<20	88 (100.0) ᵃ	413 (72.6)ᵇ	722 (87.8)ᶜ	1,223 (82.7)	
20–49	0ᵃᵇ	122 (21.4)ᵇ	86 (10.5)ᵃ	208 (14.1)	
≥50	0ᵃᵇ	34 (6.0)ᵇ	14 (1.7)ᵃ	48 (3.2)	
Length of hospital stay (days), mean (±SD)	4.20 (4.31) ᵃ	19.93 (23.14)ᵇ	14.60 (20.77)ᶜ	16.03 (21.49)	<0.001
Inhalation injury, *n* (%)					<0.001
Yes	1 (1.1)ᵃ	50 (8.8)ᵇ	5 (0.6)ᵃ	56 (3.8)	
No	87 (98.9)ᵃ	519 (91.2)ᵇ	817 (99.4)ᵃ	1,423 (96.2)	
Intubation need, *n* (%)					<0.001
Yes	1 (1.1)ᵃ	57 (10.0)ᵇ	29 (3.5)ᵃ	87 (5.9)	
No	87 (98.9)ᵃ	512 (90.0)ᵇ	793 (96.5)ᵃ	1,392 (94.1)	
Country of residency *n* (%)					0.001
Turkey	73 (83.0)ᵃ	451 (79.3)ᵃ	689 (83.8)ᵃ	1,213 (82.0)	
Immigrant/refugees	15 (17.0)ᵃ	80 (14.1)ᵃ	115 (14.0)ᵃ	210 (14.2)	
Referred from abroad	0ᵃᵇ	38 (6.7)ᵇ	18 (2.2)ᵃ	56 (3.8)	
Mortality (Exitus), *n* (%)					0.021
Yes	0ᵃ	18 (3.2)ᵃ	11 (1.3)ᵃ	29 (2.0)*	
No	88 (100.0) ᵃ	551 (96.8)ᵃ	811 (98.7)ᵃ	1,450 (98.0)	

Categorical variables were compared using the Chi-square test.

Continuous variables were analyzed using one-way ANOVA.

* The majority of the deceased patients (24, 82.75%) had TBSA levels >20%. Of the deceased patients (5, 17.24%) with TBSA levels <20%, all belonged to group 3.

Values in the same row with different superscript letters indicate statistically significant differences in Bonferroni-adjusted pairwise comparisons. Since three pairwise comparisons were performed, the adjusted significance level was set at *p* < 0.0167. Values sharing at least one superscript letter are not significantly different.

TBSA, Total body surface area.

Patients in Group 2 had higher TBSA values than those in the other groups (*P* < 0.001). Hospital stay was also longest in Group 2 (19 days) and shortest in Group 1 (4 days). The difference in length of hospital stay among the three groups was significant (*P* < 0.001).

Inhalation injury was found to be more common in patients in Group 2 (8.8%) than in the other groups (%1.1 and 0.6). Intubation and mechanical ventilation were required at significantly higher rates in this group than in the other groups (both *P* < 0.001) (Table 1). Among the patients, 266 (18%) were foreign nationals. Of these, 210 were immigrant/refugee patients living in Turkey (in camps or houses). The remaining 56 were patients (mostly with major burns) who had been transferred to us from abroad. The number of patients transferred from abroad in Group 2 was thus higher than in the other groups (*P* < 0.001). The majority of foreign nationals were Syrian citizens (%89.5). The distribution of patients according to their nationality is shown in Table 2.

**Table 2:** Distribution of countries of origin among imigrant and referred patients.

Country of origin	Imigrant	Referred	Total
	(*n* = 210), *n* (%)	(*n* = 56), *n* (%)	(*n* = 266), *n* (%)
Syria	197 (93.8)	41 (73.2)	238 (89.5)
Palestine	1 (0.5)	6 (10.7)	7 (2.6)
Afghanistan	9 (4.3)	1 (1.8)	10 (3.8)
Somalia	1 (0.5)	4 (7.1)	5 (1.9)
Other^a^	2 (1.0)	4 (7.1)	6 (2.3)

a Other includes Iraq, Lebanon, and Cyprus.

No mortality was observed in Group 1, whereas it was 1.3% in Group 3. It was higher in group 2 (3.2%), than in the other groups. Most deaths occurred in patients with TBSA ≥20% (*n* = 24, 82.8%). Notably, all patients with TBSA <20% who died (*n* = 5, 17.2%) were in Group 3.

After Bonferroni adjustment for multiple pairwise comparisons, the observed overall differences in mortality did not remain statistically significant between any individual pair of groups. In contrast, the between-group differences in TBSA distribution, length of hospital stay, inhalation injury, and intubation requirement remained significant.

Multivariable logistic regression analysis showed that burn-region involvement was not independently associated with mortality (overall *p* = 0.634). Specifically, mortality did not differ significantly between patients with SFN involvement plus burns in other regions and those with burns in other regions without SFN involvement (aOR, 0.59; 95% CI, 0.20–1.75; *p* = 0.339). In contrast, intubation (aOR, 29.44; 95% CI, 9.07–95.58; *p* < 0.001) and TBSA ≥50% (aOR, 29.28; 95% CI, 8.00–107.18; *p* < 0.001) were independently associated with increased odds of mortality. TBSA of 20%–49% and inhalation injury were not independently associated with mortality (Table 3).

**Table 3:** Multivariable logistic regression analysis of factors associated with mortality.

**Variable**	**OR**	**95% CI**	***p* value**
Intubation			
Yes	29.44	9.07–95.58	<0.001
Inhalation injury			
Yes	0.84	0.25–2.85	0.784
**TBSA**			<0.001*
20%–49%	3.31	0.95–11.50	0.06
≥50%	29.28	8.00–107.18	<0.001
Burn-region involvement			0.634*
SFN only	NE^a^	—	—
SFN + other regions	0.59	0.20–1.75	0.339

Reference categories were no intubation, no inhalation injury, TBSA <20%, and burns involving other regions without SFN involvement.

OR, adjusted odds ratio; CI, confidence interval; TBSA, total body surface area; SFN, scalp, face, and neck; NE, not estimable.

* Overall *p* value for the categorical variable.

a The odds ratio could not be reliably estimated because no deaths occurred in the SFN-only group.

The relationship between sex and age in patients with SFN burns (Groups 1 and 2, *n* = 657) is shown in Table 4. There was no difference in the sex ratio in patients under 10 years of age. However, in the adolescents, upon *post hoc* Bonferroni correction, the proportion of male patients was higher than in the younger age groups (*P* < 0.001).

**Table 4:** Distribution of gender according to age groups in pediatric patients with SFN burns.

Gender	<1 year	1–3 years	4–5 years	6–10 years	11–18 years	Total	*p* value
	(*n* = 85)	(*n* = 265)	(*n* = 102)	(*n* = 66)	(*n* = 139)	(*n* = 657)	
Gender *n* (%)							<0.001
Male	46 (54.1)ᵃ	158 (59.6)ᵃ	63 (61.8)ᵃ	38 (57.6)ᵃ	119 (85.6)ᵇ	424 (64.5)	
Female	39 (45.9)ᵃ	107 (40.4)ᵃ	39 (38.2)ᵃ	28 (42.4)ᵃ	20 (14.4)ᵇ	233 (35.5)	

Percentages are presented as column percentages. Categorical variables were compared using the Chi-square test.

Different superscript letters (a, b) indicate statistically significant differences between age groups (Bonferroni-adjusted *post hoc* test).

Scald injuries were the predominant etiology among patients with SFN burns (Groups 1 and 2) (*n* = 389, 59.2%), followed by flame burns (*n* = 215, 32.7%) and other etiologies (*n* = 42, 6.4%) (Table 5). When analyzed by age group, this order varied among the groups. Specifically, scald burns were most common in children under 5 years old, while in older children flame burns were the most common cause, similar to adults, with the difference being significantly different (chi-square, *P* < 0.001). In pairwise comparisons between groups with Bonferroni correction, scald burns were found to be significantly more common in those aged 1–3 years than in adolescents (*p* = 0.001). In contrast, flame burns were rarely seen in those aged <1 year (infants) but were very common in those aged 11–18 years (82.7%) (*P* < 0.001).

**Table 5:** Distribution of burn etiology, mechanism, and gender by Age groups in pediatric patients with SFN burns.

	<1 year	1–3 years	4–5 years	6–10 years	11–18 years	Total	*P* value
	(*n* = 85)	(*n* = 265)	(*n* = 102)	(*n* = 66)	(*n* = 139)	(*n* = 657)	
Burn etiology, *n* (%)							<0.001
Scald	63 (74.1)ᵃ	237 (89.4)ᵇ	58 (56.9)ᶜ	21 (31.8)ᵈ	12 (8.6)ᵉ	391 (59.5)	
Flame	6 (7.1)ᵃ	16 (6.0)ᵃ	40 (39.2)ᵇ	36 (54.5)ᵇ	115 (82.7)ᶜ	213 (32.4)	
Electrical	0 (0.0)	0 (0.0)	0 (0.0)	1 (1.5)ᵃ	10 (7.2)ᶜ	11 (1.7)	
Other*	16 (18.8)ᵃ	12 (4.5)ᵇ	4 (3.9)ᵇ	8 (12.1)ᵃ	2 (1.4)ᵇ	42 (6.4)	
Injury mechanism, *n* (%)							<0.001
Kitchen related	64 (75.3)ᵃ	233 (87.9)ᵇ	58 (56.9)ᶜ	26 (39.4)ᵈ	16 (11.5)ᵉ	397 (60.4)	
Heating related (stove)	14 (16.5)ᵃ	18 (6.8)ᵇ	12 (11.8)ᵃᵇ	14 (21.2)ᵃ	55 (39.6)ᶜ	113 (17.2)	
Flammable material related	0 (0.0)ᵃ	2 (0.8)ᵃ	9 (8.8)ᵇ	8 (12.1)ᵇ	35 (25.2)ᶜ	54 (8.2)	
Explosion related	4 (4.7)ᵃᵇ	7 (2.6)ᵇ	14 (13.7)ᶜ	7 (10.6)ᵃᶜ	14 (10.1)ᵃᶜ	46 (7.0)	
Other**	3 (3.5)ᵃᵇ	5 (1.9)ᵇ	9 (8.8)ᵃᶜ	11 (16.7)ᶜ	19 (13.7)ᶜ	47 (7.2)	

Values are presented as *n* (%), with column percentages. Categorical variables were compared using the chi-square test. *Post hoc* pairwise comparisons were performed using Bonferroni correction. Statistical significance was set at *p* < 0.004 for burn etiology and *p* < 0.003 for injury mechanism. Different superscript letters (a–e) indicate statistically significant differences between age groups.

* Other etiologies include chemical, contact, radiation, lightning, frostbite, and sunburn injuries.

** Other mechanisms include electrical, lightning-related, frostbite, and sunburn injuries.

The relationship between age groups and injury mechanisms is shown in Table 4. Kitchen-related burns were the most common (*n* = 397, 60.4%), reaching a rate of 75.3% in children under 1 year old and peaking at 87.9% in those aged 1–3 years. The incidence then decreased with age, dropping to only 11.5% of those aged 11–18. In older children (11–18 years), the rate of flame burns was found to be higher (39.6%) than in the other groups, while burns caused by flammable material were also much more common in this age group than in the others (*P* < 0.001). Burns related to flammable material comprised injuries associated with butane gas, volatile substances (including inhalant-type agents), alcohol-based products (e.g., cologne) and igniting devices such as lighters. Explosion-related burns were more common in those aged 4–5 years (*n* = 14, 13.7%; *P* < 0.001). These patients had suffered burns as a result of bombing during the civil war in Syria or Palestine or accidental explosions (e.g., from gas cylinders, stoves) in houses and tents. These findings show that kitchen-related burns are more common in early childhood (<10 years) and adolescence (11–18 years), with more risky mechanisms such as heating systems and flammable materials.

## 4 Discussion

Anatomically, the SFN region is at relatively high risk of burn injury and represents one of the most frequently involved parts of the body in burn patients. Such burns have serious consequences, both cosmetically and functionally (4, 5).

This study highlights the clinical heterogeneity of pediatric SFN burns. A key finding was the marked difference in burn severity and clinical outcomes between burns limited to the SFN and SFN burns with involvement of additional body regions. Although crude mortality was higher among patients with SFN burns involving other body areas, multivariable analysis showed that SFN involvement was not independently associated with mortality. Instead, greater TBSA and intubation were the main independent predictors of mortality. Isolated SFN burns were generally less extensive and more commonly associated with scald injuries, whereas SFN burns involving additional body regions were more frequently associated with flame injuries, inhalation injury, intubation, and greater TBSA. These findings suggest that the poorer outcomes observed in patients with combined SFN and additional body-region burns are primarily related to the overall extent and severity of the burn injury rather than SFN involvement. Therefore, when evaluating SFN burns, the presence of burns in other areas and the mechanism by which the burn occurred should be considered. Specifically, the coexistence of burns in other areas along with SFN burns will increase TBSA, inevitably leading to increased morbidity and mortality (3, 5, 13). In line with this, in our study, patients with SFN burns as well as burns in other regions (Group 2) had higher rates of LOS, need for intubation and mortality than patients with SFN burns alone (Group 1).

The fact that all isolated SFN (Group 1) burns affected <20% TBSA and that no such cases led to mortality suggests that TBSA, rather than anatomical localization, is the primary determinant of prognosis. In contrast, 21.4% of patients in Group 2 had TBSA between 20% and 50%, and 6% had ≥50%. The fact that the majority of patients who died (*n* = 24, 82.75%) had burns exceeding 20% TBSA, highlights the correlation between TBSA and mortality. This aligns with the literature showing that TBSA is a factor that independently affects mortality (3, 13).

Among adult burn patients, the rate of SFN burns ranges from 35% to 65% (5, 14). Such variations among studies may be explained by various factors. For example, some studies were conducted only on adult patients, while others covered all age groups. In addition, cultural differences, variations in healthcare systems, and the number of pediatric patients included in the studies could be explanatory factors. One study conducted by Tian et al., and Ngaage et al. which included patients of all ages, found that the rate of head and facial burns was 65.63% and %49 (15, 16). While some studies have evaluated SFN burns only in children, our literature search did not reveal any studies that separately evaluated isolated SFN burns and SFN burns associated with burns in other anatomical regions. In our study on pediatric patients at a Turkish burn center, SFN burns constituted 44.4% of all burns.

As with other traumas, burns are approximately twice as common in males than in females for both adults and children. In children, the proportion of males was between 55.2% and 62.97% (17–19). Similarly, in children with SFN burns, the proportion of males was between 57.3% and 69.6% (20–22). Consistent with this, in our study, the proportion of male patients was 61.5% for all burns and 64.5% for SFN burns (groups 1 and 2).

Parts of the Middle East continue to be devastated by war. As a consequence, large numbers of imigrants and refugees residing in Türkiye, both from adjacent countries (especially Syria and Iraq) and from countries with strong links to Türkiye (Palestine, Afghanistan and Somalia). According to official data from the United Nations High Commissioner for Refugees (UNHCR), Turkey is among the countries hosting the largest number of refugees globally. In fact, Türkiye hosts approximately 3.2 million registered Syrian refugees, as well as around 222,000 people of other nationalities (23). In addition, because the healthcare system in Syria has collapsed, a large number of patients with severe burns are being referred to our centre from the north of the country. We thus divided foreign patients into two groups in this study: 1-immigrants, refugees and asylum seekers residing in Turkey; and 2-those referred from their home countries after being injured in fires. Overall, foreign nationals constituted 18% of our patients. More than 14% of these were foreigners residing in Türkiye while approximately 4% were severely burned patients referred from abroad (mostly from Syria). This is because the majority of our international patients come from war zones such as Syria, and patients from war zones are often referred due to severe burns caused by bomb blasts. These severely burned patients still pose a significant burden in our country and in other countries bordering Syria (24).

Studies show that the majority of burns in infancy, particularly scald burns, are kitchen related. In some studies, this rate was as high as 70% (10, 18, 20). In our study, when considering the causes of burns according to age, scald burns accounted for 60.9% of all SFN burns, whether they were accompanied by other burns, and this rate was significantly higher in infancy. Similarly, the rate of kitchen-related burns was found to be high. Among such burns, the most common causes were boilig water and hot beverages. The high incidence of scald burns in infancy can be explained by insufficient motor coordination, lack of awareness of danger, developmental curiosity and the need for parental supervision (13, 25, 26). Keeping young children away from the kitchen, turning pot and kettle handles inwards, not leaving hot liquids near countertops, stoves or ovens, not letting kettle cords hang down, keeping hot drinks out of reach of children and increasing caregiver awareness are standard measures for reducing the incidence of burns (27–29).

School-age children are characterised by increased mobility and exposure to a more diverse range of environments. The literature also reports that the range of causes of burns expands with increasing age in pediatric patients (30). Conversely, SFN burns caused by flames are significantly more common in adolescents, which is consistent with the literature (8). Electrical burns were also more common in this age group. Reasons for this include the increased autonomy of adolescents, as well as them spending more time outdoors and engaging in risky behaviours.

Our study also showed that burns caused by flammable materials predominantly affect adolescents. Gaseous inhalants with the potential for abuse and heating-related sources were the main causes of flame burns in adolescents. Butane gas, in particular, is readily available due to its use in many products such as paint, shoe polish, adhesive and lighter fluid, its presence in most homes, its low cost and the lack of legal restrictions. In this context, the number of lighter fluid addicts is increasing; consequently, cases of burns associated with this are on the rise. As shown in our study, burns are predominantly suffered by males. It was previously reported that all lighter fluid burns involve the face, while associated inhalation burns have also been reported to be common (31, 32).

Face and neck burns carry a high risk of the development of upper airway mucosal oedema, airway damage and inhalation injury due to carbon monoxide exposure (5, 33, 34). In burn patients, inhalation injury has been reported to increase mortality, along with TBSA and age (35). In this study, the rate of inhalation injury was higher in patients in Group 2 than in the other groups, which is consistent with the literature.

Our study has some limitations, which should be mentioned here. The first is the retrospective nature of this study. Another limitation is that this work involved only a single centre. Prospective and multicentre studies can yield more meaningful results on this subject. Another limitation of our study is that only inpatients were included. The inclusion of outpatients could potentially alter the data on outcomes, particularly mortality. Therefore, we are considering the inclusion of outpatients in future studies.

Pediatric scalp, face, and neck burns should not be considered a single uniform category. Isolated SFN burns were generally limited in extent, whereas SFN burns involving additional body regions were associated with greater overall burn severity and higher crude rates of inhalation injury, intubation, and mortality. However, multivariate analysis showed that SFN involvement itself was not independently associated with mortality, whereas a greater TBSA was a significant predictor of mortality. These findings suggest that the poorer outcomes observed in patients with SFN burns with involvement of additional body regions are primarily related to the overall extent and severity of the burn injury rather than SFN involvement itself.

These findings demonstrate that early airway assessment, serial clinical examination and early intubation in patients with suspected inhalation injury are critically important, especially in the presence of concomitant extensive TBSA in cases of SFN burns. Providing training on this subject to Turkish healthcare professionals who provide initial treatment and intervention to pediatric burn patients is becoming increasingly important.

## Data Availability

The raw data supporting the conclusions of this article will be made available by the authors, without undue reservation.
